# Balancing the brain of offenders with psychopathy? Resting state EEG and electrodermal activity after a pilot study of brain self-regulation training

**DOI:** 10.1371/journal.pone.0242830

**Published:** 2021-01-07

**Authors:** Lilian Konicar, Stefan Radev, Stefano Silvoni, Elaina Bolinger, Ralf Veit, Ute Strehl, Christine Vesely, Paul L. Plener, Luise Poustka, Niels Birbaumer

**Affiliations:** 1 Department of Child- and Adolescence Psychiatry, Medical University of Vienna, Vienna, Austria; 2 Institute for Medical Psychology and Behavioural Neurobiology, Eberhard-Karls-University of Tübingen, Tübingen, Germany; 3 Institute of Psychology, University of Heidelberg, Heidelberg, Germany; 4 Department of Cognitive and Clinical Neuroscience, Central Institute of Mental Health, Medical Faculty Mannheim, Heidelberg University, Mannheim, Germany; 5 Institute for Diabetes Research and Metabolic Diseases of the Helmholtz Center Munich at the University of Tübingen, Tübingen, Germany; 6 Department of Child-& Adolescence Psychiatry and Psychotherapy, Medical University of Göttingen, Göttingen, Germany; 7 Wyss Center for Bio and Neuroengineering, Geneva, Switzerland; University of Graz, AUSTRIA

## Abstract

Although investigation of the brains of criminals began quite early in the history of psychophysiological research, little is known about brain plasticity of offenders with psychopathy. Building on our preliminary study reporting successful brain self-regulation using slow cortical potential (SCP) neurofeedback in offenders with psychopathy, we investigated the central nervous and autonomic peripheral changes occurring after brain self-regulation in a group of severe male offenders with psychopathy. Regarding the central nervous system, an overall suppression of the psychopathic overrepresentation of slow frequency bands was found, such as delta and theta band activity, after EEG neurofeedback. In addition, an increase in alpha band activity could be observed after the SCP self-regulation training. Electrodermal activity adaptively changed according to the regulation task, and this flexibility improved over training time. The results of this study point towards a constructive learning process and plasticity in neural and peripheral measures of offenders with psychopathy.

## 1 Introduction

Individuals with psychopathy are characterized by a lack of empathy, anticipatory fear and remorse after criminal acts, as well as shallow affect in combination with elevated behavioral approach tendencies including heightened aggressiveness and anger [1–5]. One of the most established and widely used diagnostic tools for measuring psychopathy is the Hares’ Psychopathy-Checklist-Revised (PCL-R) [1], overcoming pitfalls of self-assessment scales for the measure of psychopathic personality. Several large PCL-R studies [1, 6–8] confirmed Hare’s model of psychopathy, including (a) the *Interpersonal Facet* (describing a superficially charming, yet cunning and manipulative person), (b) the *Affective Facet* (which describes shortcomings such as lack of empathy and remorse, as well as limited emotionality), (c) the *Lifestyle Facet* (which captures characteristics such as need for stimulation, impulsivity, and sensation seeking) and (d) the *Antisocial Behavior Facet* (which encompasses poor behavioral control, criminal versatility and conflicts with criminal law).

Moreover, the first two facets, the core psychopathy features (the *Interpersonal Facet* and *the Affective Facet*) could be condensed as the factor 1 (F1), whereas the factor 2 (F2) covers *the Lifestyle Facet* and the *Antisocial Behavior Facet* [1], which is closely related to the construct of Antisocial Personality Disorder. This conceptualization makes it clear, that an antisocial feature is inherent in every individual with psychopathy according to the PCL-R, but not *vice versa* (e.g., not every individual with antisocial personality disorder displays F1 core psychopathic features). The two psychopathic factors (F1+F2) were found to be differently associated with psychological, behavioral and peripheral parameters with potentially distinct etiologies [9]. Therefore, it is of great importance to disentangle these facets in order to provide a more precise and specific description of the disorder.

In addition to clinical descriptions of the personality and behavior of individuals with psychopathy, the investigation of biological abnormalities in offenders and antisocial individuals has a long history. Already in 1938, Jasper and colleagues [10] published the first EEG results reporting differences in the brainwaves of children with behavior problems. Later, Hill and Pond [11], Kennard [12] as well as Bach-y-Rita and colleagues [13] and others continued studying the brains of offenders, antisocial individuals and violent patients, demonstrating a slowing of the EEG that is primarily expressed over temporal regions. In the following years, methodological limitations in these earlier studies were overcome by EEG studies with improved study designs, automatic signal processing and advanced feature extraction [14–30].

The most common finding in these studies of antisocial individuals supports the early findings of excessive slow-wave activity in the EEG. In this more recent body of work, slow frequencies are limited to a 1–8 Hz range, and abnormal activation is generally found at temporal and/or parietal regions. Abnormal amounts of slow frequency activity, such as delta (0.5–3.5Hz) and theta frequency band (3,5–7,5Hz), are not only linked to decreased arousal [31], but also to elevated anger, aggressiveness and violence [e.g. 16], which are hallmarks of antisocial personality. Recently, a review [9] summarizing the latest EEG findings shows another picture of individuals with psychopathy. Based on numerous findings of Event-Related-Potential (ERP) studies, the results of this review point toward normal cognitive functioning of individuals exhibiting pronounced psychopathic traits (inmates and offenders, as well as community samples and undergraduate samples were comprised in this review), while emotional functioning seems nevertheless to be especially disturbed.

On the other hand, research regarding resting state EEG of individuals with psychopathy (in contrast to the abundant EEG research in antisocial individuals) is very rare. Besides spectral power analyses of the EEG during different tasks [31–36], until now only one recent study investigated resting state EEG in delinquents with psychopathy. Using EEG and Low-Resolution Brain Electromagnetic Tomography (LORETA), Calzada-Reyes and colleagues [15] found a widespread slow EEG pattern in offenders with psychopathy, similarly to the slow activity increase in antisocial offenders without psychopathy. Although increases in beta activity were found only in the offender group with psychopathy and linked to the psychopathy-related superficial charm by the authors, the differential topographic level of beta are ambiguous and are therefore not discussed in the following. A more robust finding is the reported decrease of alpha frequency power at midline, parietal and temporal regions, only seen in the offender group with psychopathy [15]. This attenuation of alpha activity was linked to shallow affect and failure to accept responsibility for one’s own actions in the offender sample with psychopathy.

Furthermore, the reduced power in the alpha frequency band (8–12 Hz) seems to be also one of the most robust findings (besides the excessive slowing of EEG activity) in individuals with behavioral and antisocial disorders [14, 16, 19, 27, 37, 38]. Besides that alpha frequency is defined as a general marker of inhibition [39, 40], alpha frequency band is also said to represent long-range communication between different brain regions [41]. Considering the dysfunction of the alpha frequency band in individuals with psychopathy, it is therefore no wonder that especially the disrupted communication between different brain areas is said to be one of the neuroanatomical keys to antisocial and violent individuals, as well as to individuals with psychopathy [42, 43].

This notion is supported by neuroimaging studies using Magnetic Resonance Imaging (MRI) in individuals with antisocial personality disorder or with psychopathy. Structural brain deviations were reported in frontal areas including the ventromedial, orbitofrontal and dorsolateral prefrontal cortices as well as in subcortical and limbic areas, such as the amygdala, insular cortex, striatum, hippocampus and anterior cingulate cortex [43–52]. Kiehl and colleagues [49] proposed that deficits within a paralimbic network ranging from the orbito-frontal cortex to the posterior cingulate cortex and temporal lobe play a crucial role in the development and persistence of psychopathy.

Regarding physiological biomarkers of individuals with antisocial or psychopathic characteristics, many studies have investigated indices of electrodermal activity (EDA, e.g., skin conductance or its inverse, skin resistance). Extensive evidence has confirmed reduced skin conductance at rest, in anticipation of an aversive event, or as a reaction to a stimulus in individuals with antisocial and/or psychopathic characteristics [2–5, 53–58].

Although scientific evidence increasingly suggests that there is a distinct neurobiological basis of psychopathy, research targeting neurobiologically based treatment approaches for this group of individuals is still in its infancy. Until now, only one study [59] conducted a neurobiological treatment in the form of an EEG-based neurofeedback training based on Slow Cortical Potentials (SCPs) for offenders with psychopathy. This study investigated whether offenders with psychopathy are generally able to gain control over their brain activity. The task of the participants with psychopathy was to move a cursor on the feedback screen upwards or downwards by controlling the polarity of their SCPs, that is, by learning to volitionally produce electrically slow negative shifts (move cursor upwards by increasing cortical excitability in the *negativity task*) or slow positive shifts (move cursor downwards by decreasing cortical excitability in the *positivity task*). The results of this study showed that offenders with psychopathy successfully learned to volitionally produce negative and positive SCP shifts and further increased the differentiation between these two brain states (neuroelectric negativity/ positivity). Furthermore, the authors reported [59] that offenders with psychopathy, displayed decreases in self-reported aggression and self-reported behavioral approach, which occurred in parallel to an increase in behavioral inhibition and cortical activity / sensitivity to failures and errors.

However, not a single study has shed light on the highly interesting brain-body connection link that underlies brain regulation and its potential modification in individuals with psychopathy. Based on the pioneering work in [59], we aim to pave the way for more integrative research approaches and, further, to explore peripheral-physiological parameters after brain self-regulation. By using the data from Konicar et al. [59], the aims of the current study were the following.

At the neural level, we sought to expand the rare EEG research in psychopathy and investigated whether the summarized findings of EEG slowing in antisocial individuals, as well as the reduced amount of alpha frequency, could also be confirmed in our highly psychopathic sample at baseline (e.g., in the data of Konicar [59]). Secondly, we examined whether there are detectable changes in EEG activity as measured before and after SCP-neurofeedback training. In line with the literature, we hypothesized that the overrepresentation of slow frequency bands would be decreased, while alpha band activity would be increased after brain regulation training at parietal-temporal and midline sites. In addition, it was of interest to determine how these possible EEG changes from pre- to post SCP-training are related to psychopathy and its different facets as well as to the indices of the SCP-neurofeedback training itself.

At the peripheral level, we expected that the pattern of changes in electrodermal activity during the course of SCP training would be in line with the observed pattern of changes in cortical activity. Thus, we expected an increase in skin resistance during brain regulation trials requiring a reduction of cortical activity (positivity trials), while a decrease in skin resistance during brain regulation trials requiring an increase of cortical activity (negativity trials) was expected. Accordingly, we also anticipated an increase in differentiation (i.e., a larger difference between EDA in positivity and negativity trials) during the course of SCP training.

Lastly, we investigated the relationships between cortical and peripheral changes and changes in subjective self-reports (e.g., aggression, empathy, behavioral approach) in an exploratory manner. Due to the purely exploratory nature of these relationships, these analyses, together with additional analyses regarding another peripheral physiological parameter—heart rate activity—are provided in the S1-S4 Sections in S1 File.

## 2 Materials and methods

### 2.1 Experimental design

The present report is part of a larger pre/post-multilevel-cross-validated intervention study using the same psychopathic sample as in the already published results of the main study (Konicar et al. [59]). The main study investigated if severe offenders with psychopathy were able to gain control of their frontal brain activity and if this improved self-regulation affects psychopathic traits on the subjective, behavioral and cortical level. The authors reported an increase in SCP-differentiation at the end of the training, in parallel decreases in self-reported aggression and self-reported behavioral approach. Furthermore, they showed an increase in behavioral inhibition as well as an increase in cortical activity / sensitivity to failures and errors after SCP-training [59]. The reported concordant changes indicate successful brain self-regulation, even considering the lack of a control group in this single group pre-post design (for justifications of the design see S5 Section in S1 File).

The current study was approved by the ethical committee of the Faculty of Medicine of the University of Tübingen, Germany according to the Declaration of Helsinki. Written informed consent was obtained from each participant regarding all measurements.

### 2.2 Participants

The same sample of criminal patients with psychopathy as in the main study (Konicar et al. [59]) was used for the extended analysis of this report. Participants were recruited in high-security forensic-psychiatric clinics in Germany. Inclusion criteria included an age of 18 years or older, as well as a classification of a psychopathic personality (Psychopathy-Checklist-Revised, PCL-R [1]). Patients with an IQ below 80 (on CFT 20-R [60]), neurological and medical illnesses or head injuries, as well as patients with major Axis I diagnosis of psychosis, obsessive-compulsive disorder, tics or Tourette- syndrome were excluded. None of the patients received antipsychotic or sedative medication. In agreement with the forensic institutions, offenders received financial compensation of 100€ in total for all experiments.

The sample consisted of 14 male patients (mean age: 43.14 ± 11.52 years, all right handed) with a long history of criminal acts related to grave offences, serving long-term sentences. Convictions were related to serious violent behavior such as murder, violent robbery, blackmail, dangerous assault with weapon, attempted manslaughter and/or severe sex crimes such as repeated sexual assault, rape, and necrophilia. All of the participants committed more than one (mostly 3–7 different) serious infringements and were sentenced to involuntary inbound commitment in high security psychiatric-forensic facilities for containment of danger/preventive detention. Based on the Psychopathy Checklist-Revised (PCL-R) Interview [1] and on the current clinical status, only subjects with a PCL-R score of ≥ 26 were accepted in the study, which is above the German and European proposed cut-off score [61]. The final sample scored 30.14 points on the PCL-R on average (range: 26–34), which reflects a highly psychopathic European study sample. Because there exists no control sample, which would specifically match these exceptional subjects’ history, a within-subject design was chosen and only correlative results of this group of offenders with psychopathy are presented without any claim of causal inferences between the investigated variables (for further discussion see S5 Section in S1 File).

### 2.3 Brain self-regulation intervention: Slow cortical potential neurofeedback

**Fig 1:**
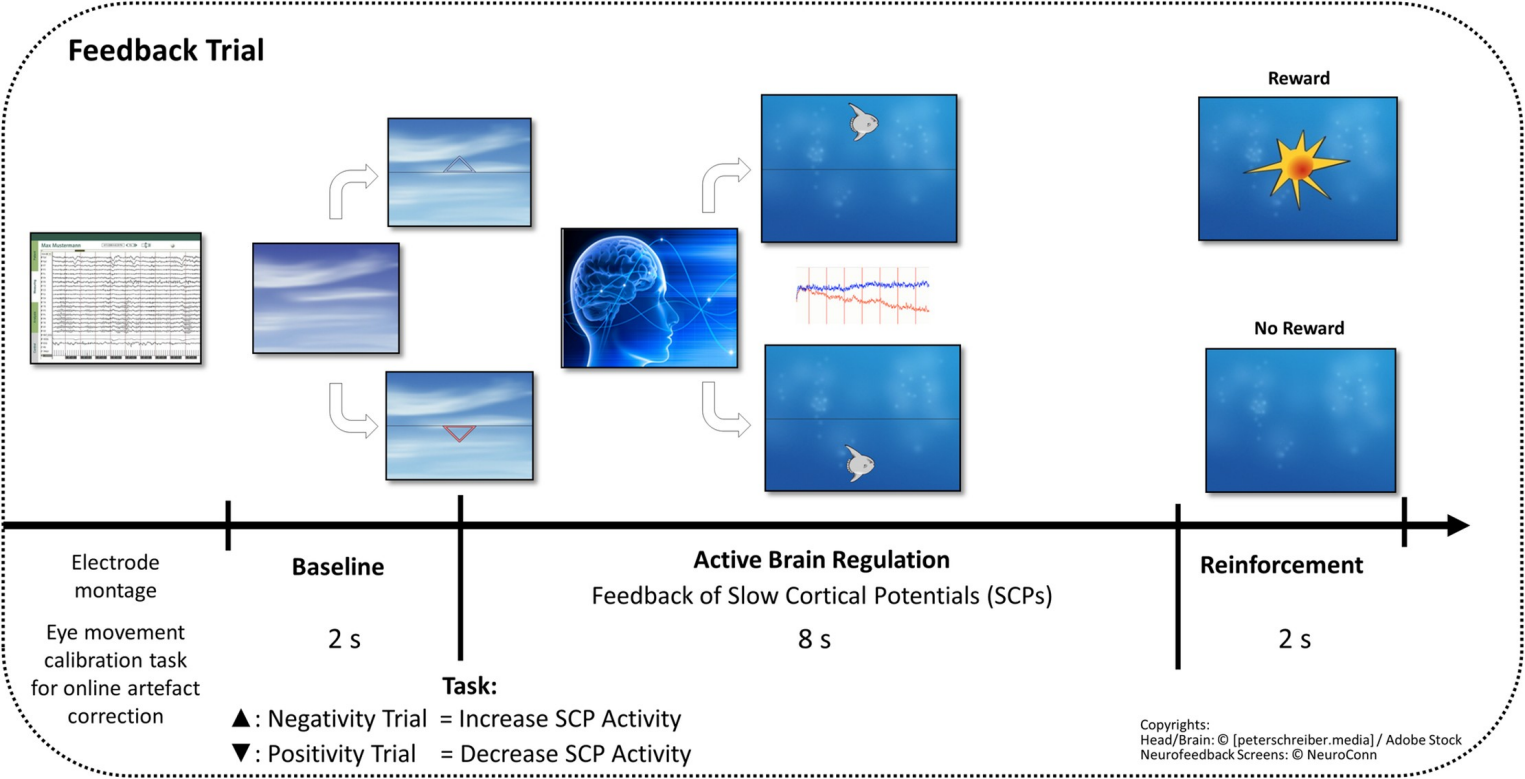
SCP neurofeedback procedure.

All participants underwent 25 EEG-based Slow Cortical Potentials (SCP) neurofeedback training sessions. SCPs were recorded at fronto-central regions and fed back to the patients’ monitor matched to preferences of the participants (e.g. fish, moon etc.). At the beginning of each trial, a triangle was displayed, specifying the polarity of the requested SCP shift of the upcoming regulation trial: a triangle pointing upwards required negative SCP shifts (increase of cortical activation), while a triangle downwards indicated required positive SCP shifts (inhibition of cortical activation). In the active regulation phase, the current SCP-activity was displayed as an object (e.g. a fish or a moon) at the participant’s screen in real time and moved accordingly to the participants brain activity upwards (indicating an increase in cortical activation) or downwards (indicating a decrease in cortical activation). The participants were required to learn how to volitionally move the object up- or down by controlling their SCPs in the required polarity. No specific strategy was suggested; subjects were instructed to develop their individual strategy to move the objects. The neurofeedback instruction emphasized that muscular (i.e. tension-relaxation) or respiratory strategies disturb self-regulation performance. Successful changes in cortical activity were rewarded with the symbol of a sun after each trial, as depicted in Fig 1.

Each neurofeedback training session consisted of three regulation blocks: starting with a *feedback block*, followed by a *transfer block* (no brain activity was fed back to the participant’s monitor, just a reward was displayed after a successful trial to facilitate the transfer of learned skill into daily life) and ended with a second *feedback block*. Two tasks, a negativity task (required production of electrically negative SCP shift; 50% in the first; 80% in the second training phase) and a positivity task (required production of electrically positive SCP shifts; 50% in the first; 20% in the second training phase) were presented in random order. Besides the visual inspection of the online data at the Theraprax system monitor in real time, direct visual observation of the participant (chest, stomach, legs, fingers, head etc.) was conducted by two experienced trainers and participants were corrected if necessary. EEG shifts larger than 200μV, eye- and movement artifacts were detected automatically online, during the SCP-training by the Theraprax System with an online artifact correction. A trial with an artifact immediately stopped and repeated.

During all SCP-neurofeedback sessions, electrodermal response was measured in parallel, which is one focus of the current study.

**Fig 2:**
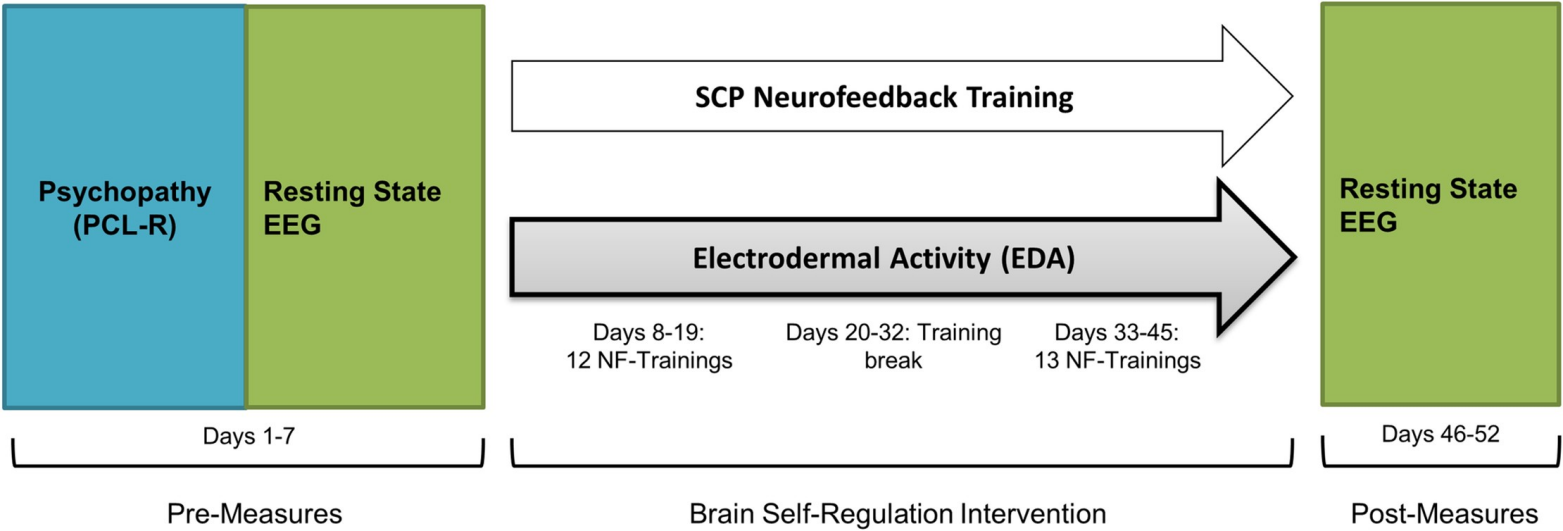
Study overview. Initial clinical assessment: psychopathy (blue); Pre-/Post-measures: resting state EEG (green); Intervention: SCP-neurofeedback training; Measures during intervention: (a) Cortical measure: SCP activation (white), (b) Peripheral-physiological measures: Electrodermal activity (grey). SCP training consisted of 25 training sessions, each split into three training blocks with different conditions (feedback–transfer–feedback) and tasks (negativity–positivity) of 8 minutes per block.

An overview of the timing aspects of the central and peripheral psychophysiological measures is given in Fig 2.

### 2.4 Psychopathy measures

Psychopathy was measured using the Psychopathy-Checklist-Revised [1]. Based on a semi-structured clinical interview in combination with the review of collateral information such as criminal records, 20 items regarding psychopathy should be scored, belonging to the four facets: The *Interpersonal Facet*, the *Affective Facet*, *the Lifestyle Facet* and the *Antisocial Behavior Facet*. The Psychopathy-Checklist-Revised interview was conducted once with each participant separately, as depicted in Fig 2.

### 2.5 Resting state EEG measures and analyses

Cortical, as well as peripheral physiological data were recorded using a Theraprax Q-EEG-System (NeuroConn GmbH, Illmenau, Germany). Resting state EEG was recorded individually with each criminal patient before and after SCP neurofeedback and consisted of eight 1-min trials, four with eyes open (O), four with eyes closed (C), in counterbalanced order (COCO-Break-OCOC). A total amount of 16-min trials for each patient in the two sessions were recorded (8 min pre- and 8 min post-treatment). EEG data were collected with Ag/AgCl electrodes at 19 EEG sites (Fp1, Fp2, F3, F4, F7, F8, Fz, FCz, C3, C4, Cz, T5, T6, P4, P4, Pz, O1, O2, Oz). In line with previous literature (summarized in *1*. *Introduction*), relative power estimates (e.g. areas under the curve, normalized over the total EEG power in the frequency range 0.4–30 Hz) of all targeted oscillatory indices (theta, delta and alpha band) were investigated at parietotemporal (average of T5, T6, P3, P4) and midline (average of Fz, Fcz, Cz, Pz, Oz) EEG sites.

During recording, all sites were referenced to an average mastoid reference (M1/M2). Blinks and eye movements were monitored via two ocular electrodes (vertical placement, VEOG1 and VEOG2). Electrode impedances were kept below 3kOhm throughout the study. EEG data were recorded at a sampling rate of 128 Hz. Continuous EEG data were pre-processed using the following procedure: direct current (DC) removal; band-pass filtering 0.4–30 Hz (order 3, zero-phase, elliptic filter); ocular artefacts removal using VEOG1 and VEOG2 as reference (a blind source separation algorithm was used [62, 63]). From each EEG recording half-overlapped epochs of 2 *s* length were extracted. Portions of data with artifacts were excluded from the analysis using the following rejection criterions (if at least one criterion was satisfied, the epoch was excluded): (a) max. gradient 50 μV/ms, (b) max allowed absolute difference of 200 μV in intervals of 200 *ms*, (c) max/min absolute amplitude (max 200 μV, min -200 μV), (d) low voltage below 0.5 μV in intervals of 100 *ms* length. Spectral power was estimated by Welch's method [64] for all artifact-free epochs of each EEG recording, separately for each participant for eyes closed (EC) and eyes open (EO) recording condition. On average 3.6±5.4% ($\bar{X}$ ± S.D.) of the epochs were rejected. Within each single EEG recording the spectra were averaged across all artifact-free epochs. Based on relevant literature, we focused on the relative power estimates for alpha frequency band (8.0–13.0 Hz) as well as on the slow frequency bands: delta (0.5–3.4 Hz) and theta (3.5–8.0 Hz).

### 2.6 Electrodermal activity measures and analyses

Due to the technical requirements of the neurofeedback system, electrodermal response, e.g. electrodermal activity (EDA), was recorded as skin resistance during the SCP-neurofeedback training with a sampling rate of 128 Hz from the Theraprax Q-EEG-System (neuroConn GmbH, Illmenau, Germany). Even though it is conventional to analyze electrodermal response in units of conductance (microSiemens), we refer to a previous study assessing the interchangeability of skin conductance and skin resistance [65] and therefore analyzed the recorded raw resistance data.

Electrodermal response was recorded using an EDA-sensor (TSD203), a set of two Ag-AgCl electrodes (unpolarizable; contact area 6mm dia; Dimensions: 16mm x 17mm x 8mm), which incorporate molded housings designed for finger attachment, as a part of and connected to the Theraprax Q-EEG-System (neuroConn GmbH, Illmenau, Germany). For measuring the electrodermal response via the voltage (root mean square) at the electrodes, a DC current of 1,5 μA together with an AC current of 6 μApp with a frequency of 1,3 kHz was applied. Each transducer includes a stretchable Velcro®strap for easy attachment. Ensuring good signal quality, firstly electro-conductive Ten20 paste was filled inside the cavities of the finger strip sensors at the surface of the Ag-AgCl electrodes before they were attached to the middle phalanges of the index and the ring finger of the participants with psychopathy. Participants were instructed to sit as comfortably as possible and move as little as possible, which was sufficiently feasible due to the cognitive attention on the neurofeedback process.

The skin resistance data were preprocessed as follows to provide artefact-free EDA data excluding outliers or extreme values: 1) Raw data was imported into MATLAB [66]; 2) slow, low-frequency drift was removed, applying low frequency trend removal; 3) data for each participant, session, and block was segmented into 10-second trials; 4) a raise in electrodermal response was classified as a response if its absolute value exceeded a threshold of 0.05 kΩ within the specified time window (similarly to Gao et al. [67]). A combination of automatic and visual rejection procedures was used to remove artefactual trials. Trials containing a signal higher than a threshold of 25 kΩ were automatically rejected, and an extensive visual inspection was used to reject noisy trials. 6) Trials were then averaged and baseline corrected across each training block, and further averaged across the first and last 6 sessions of the SCP-neurofeedback training period. 7) Responses were reduced to a single metric (referred to as ‘electrodermal activity’, ‘EDA’) by computing the average absolute response over a time window between 4 and 8 seconds after trial onset (as suggested by Figner & Murphy [68]).

### 2.7 Statistical analyses

To detect changes in resting state EEG, an exploratory repeated ANOVA with factors *“Time”* (pre intervention/post intervention), *“Region of interest*” (ROI: midline/parietotemporal), *“Frequency band”* (delta/theta/alpha) and *“Eyes*” (EC: eyes closed/ EO: eyes open) was computed. Following ANOVA, post-hoc tests for particularly interesting differences were applied.

In order to assess the total EDA regulation achieved throughout a given training session, the data were aggregated over the three training blocks into a total regulation score for the first six and the last six regulation sessions. A 2x2 repeated measures ANOVA with factors *“Time”* (first six training sessions / last six training sessions), and *“Task”* (positivity trials / negativity trials) was then performed on the aggregated data. In order to investigate whether the psychophysiological EDA-differentiation changed from the first six to the last six training sessions, the activity during negativity task was subtracted from the positivity task. This was done separately for the feedback and transfer blocks. A 2x2 repeated measures ANOVA with factors “*Time”* (first six sessions/ last six sessions), and “*Condition”* (feedback blocks / transfer blocks) was then performed on the differences data. In addition to statistical significance values, Bayes factors were computed and reported.

Parallel to the analysis of the main study [59], the SCP-recordings were separately averaged for the two conditions (feedback and transfer) and tasks (required positivity and negativity) for each session. The measured increase in learned SCP-differentiation (between required positivity and negativity tasks) from the beginning to the end of the SCP-training, reported in Konicar et al. [59] is based on the average of SCP amplitudes of the group of offenders with psychopathy. As an individual learning indicator the non-standardized *ß*-value of the linear regression of the SCP differentiation for each participant across training sessions, separately for both conditions, was used.

As an extension of the present analyses, an individual learning indicator for the positivity task over time (SCP positivity coefficient) and for the negativity task over time (SCP negativity coefficient) were computed in the same vein. Standard Pearson correlations were then computed between these measures.

Due to the lack of directional hypotheses regarding these analyses, no Bonferroni corrections were applied to post hoc tests and correlation coefficients. Thus, these analyses are to be regarded solely as suggestive of certain tendencies requiring further investigation in a confirmatory context. Greenhouse-Geisser correction was applied whenever the sphericity assumption was violated.

## 3 Results

### 3.1 Resting state EEG measures

#### 3.1.1 Baseline measures: Relationships between resting state EEG and psychopathy scores (t_1_)

**Fig 3:**
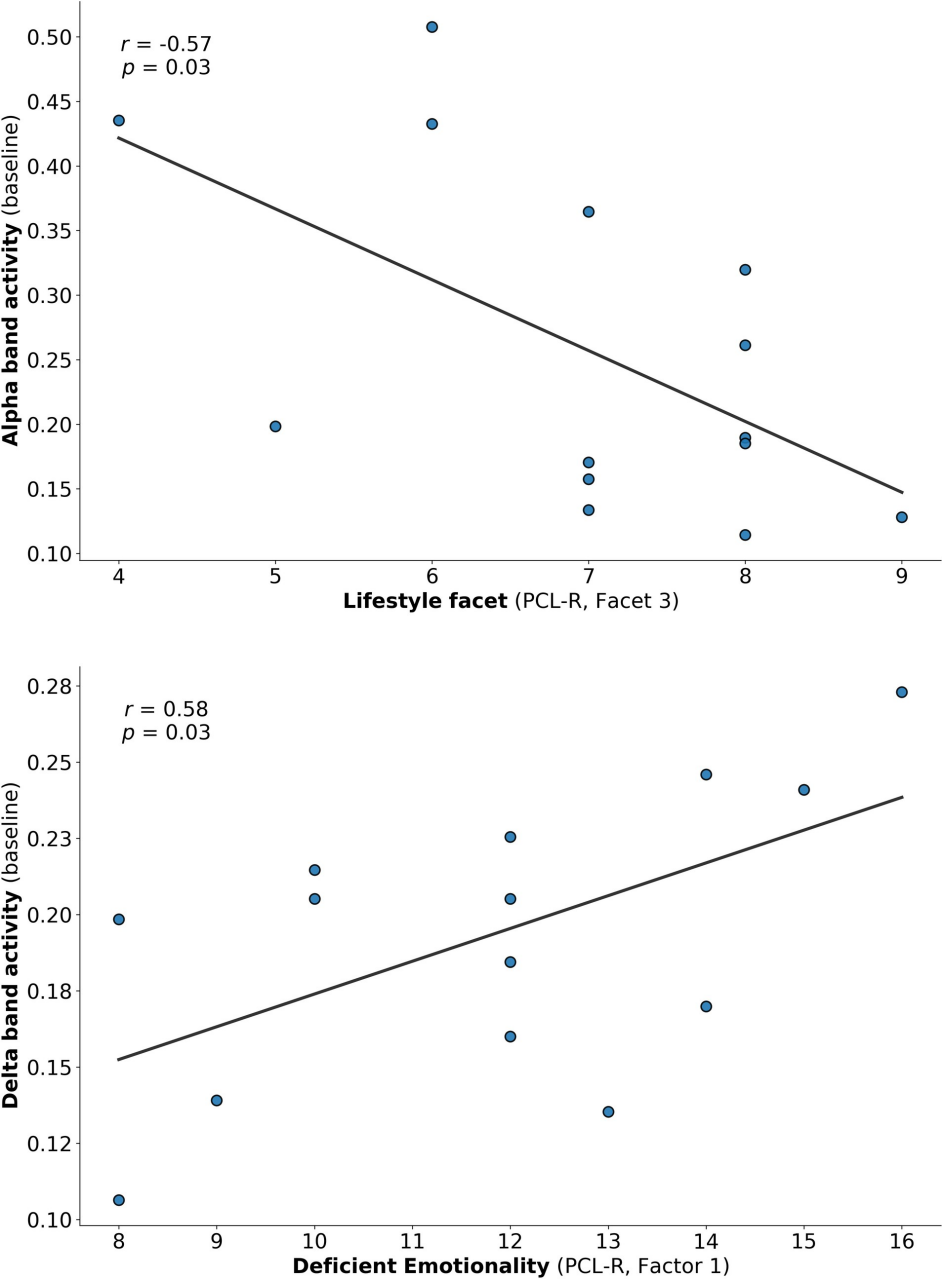
**a.** Correlation between the Interpersonal Facet (PCL-R) and resting state EEG in the delta band: The higher the interpersonal deficits, the higher the activity of delta frequency band at baseline (before the brain self-regulation training). Each dot represents one participant. **b.** Correlation between Psychopathic Lifestyle Facet (PCL-R) and resting state EEG in the alpha band. The more sensation seeking and impulsive the subject is, the lower the activity of alpha power at baseline (before the brain self-regulation training). Each dot represents one participant; bigger/bold dots represent multiple participants.

Analysis of the relationship between resting state EEG measures at baseline and psychopathy scores revealed the following pattern: the higher the participants scored on the PCL-R Total Score, the larger was their delta frequency band activity at midline sites during eyes closed (EC) condition (*r* = .634, *p* = .015). Disentangling psychopathy into the four facets of the PCL-R revealed an important role particularly for the *Interpersonal Facet*: the higher the subject score on the *Interpersonal Facet* of the PCL-R, the higher the power in delta frequency band at baseline over midline regions (EC: *r* = .548, *p* = .042) as depicted in Fig 3A.

The opposite associations were found regarding the alpha frequency band, as depicted in Fig 3B: The higher the subjects scored on the *Lifestyle Facet* (e.g. the more sensation seeking and impulsive the participant was at baseline), the lower was their alpha frequency power at midline region (EC: *r* = -.568, *p* = .034) as well as at parietotemporal regions (EC: *r* = -.572, *p* = .033) before neurofeedback intervention.

No significant relationship regarding the PCL-R Total score or the other facets of the PCL-R and resting state EEG measures were found.

#### 3.1.2 Pre-post measures: Changes in resting state EEG (t_2_-t_1_)

**Fig 4:**
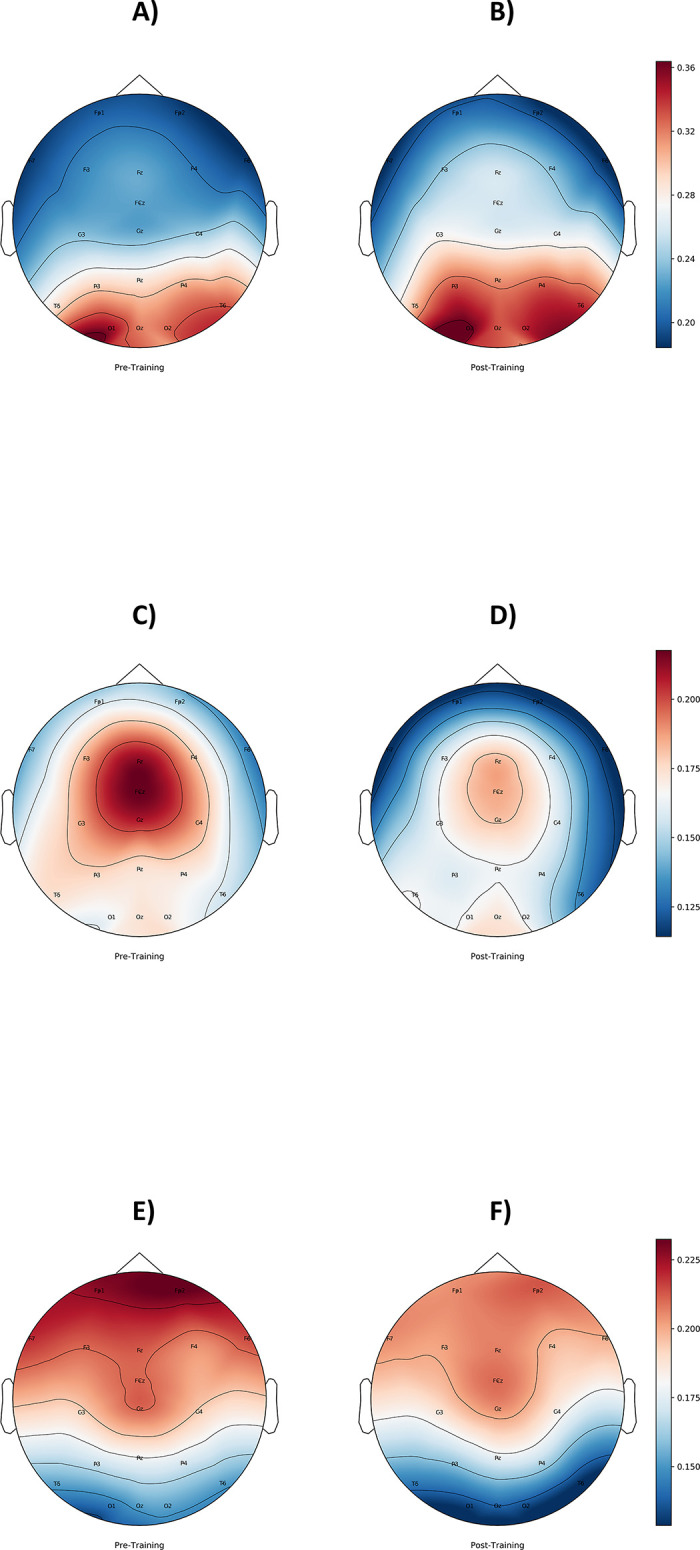


Repeated-measures analysis of variance (see Table 1) revealed a significant main effect of *‘ROI’* (region of interest) with higher power at midline sites compared to parietotemporal regions and a main effect of *‘Eyes’*, indicating higher power during the eyes closed condition than during the eyes open condition (for a topography power spectrum map of changes in resting state EEG in the eyes closed condition see Fig 4).

**Table 1:** Repeated-measures analysis of variance.

Within Subject Effect	Requ.	*df*	*F*	*p*	*η^2^*
**Frequency**	SA	2	3,108	*0*,*062*	0,193
	GG+	1,386	3,108	*0*,*084*	0,193
**Time**	SA	1	1,039	0,327	0,074
	GG	1,000	1,039	0,327	0,074
**ROI**	SA	1	14,075	0,002*	0,520
	GG	1,000	14,075	0,002	0,520
**Eyes**	SA	1	13,497	0,003*	0,509
	GG	1,000	13,497	0,003	0,509
Time* Freq	SA	2	6,012	0,007*	0,316
	GG	1,835	6,012	0,009	0,316
Time * Eyes	SA	1	2,969	0,109	0,186
	GG	1,000	2,969	0,109	0,186
Time * ROI	SA	1	2,813	0,117	0,178
	GG	1,000	2,813	0,117	0,178
Eyes * ROI	SA	1	18,120	0,001*	0,582
	GG	1,000	18,120	0,001	0,582
Freq * ROI	SA	2	48,901	0,000*	0,790
	GG	1,648	48,901	0,000	0,790
Freq * Eyes	SA	2	13,653	0,000*	0,512
	GG+	1,057	13,653	0,002*	0,512
Time * ROI* Eyes	SA	1	6,091	0,028*	0,319
	GG	1,000	6,091	0,028	0,319
Freq * ROI* Eyes	SA	2	6,254	0,006*	0,325
	GG	1,816	6,254	0,008	0,325
Freq * Time * Eyes	SA	2	0,819	0,452	0,059
	GG+	1,239	0,819	0,404	0,059
Freq * Time * ROI	SA	2	0,375	0,691	0,028
	GG	1,538	0,375	0,638	0,028
Freq * Time * Eyes * ROI	SA	2	1,313	0,286	0,092
	GG	1,650	1,313	0,285	0,092

Freq = EEG Frequency Band, Requ. = Requirement; SA = Sphericity assumed; GG = Greenhouse-Geisser Corrected; GG+ = Greenhouse-Geisser Corrected values must be used due to a violation of the Sphericity requirements.

**Fig 5:**
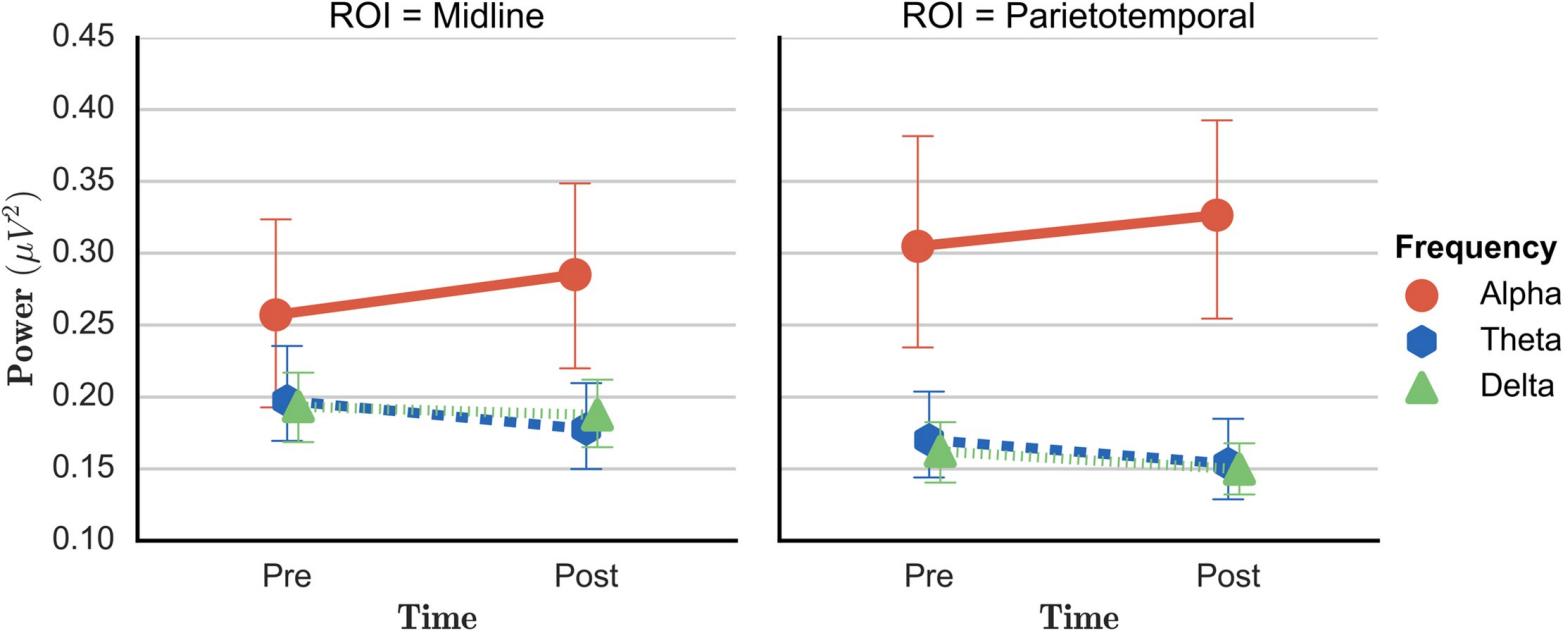
ab. Changes in resting state EEG before and after SCP neurofeedback at midline regions (5a: left) and parietotemporal regions (5b: right) during the eyes-closed condition. The y-axis values are the fraction of the total EEG power. Red line: Increase in alpha frequency band power from before to after SCP neurofeedback training. Decreases from before to after SCP neurofeedback training in delta frequency band (blue line) and theta frequency band power (green line). Vertical lines represent the standard error.

Due to significant interactions such as ‘Time*Frequency’ and ‘Time*ROI*Eyes’, pairwise post-hoc tests were conducted and revealed a significant decrease in delta frequency band during open eyes (EO: *t*_(13)_ = 3.344, *p* = .005), as well as during closed eyes condition (EC: *t*_(13)_ = 2.249, *p* = .042) in parietotemporal regions after the brain training. In line with the delta band decrease, theta band activity decreased during eyes open (*t*_(13)_ = 2.544, *p =* .024), as well as during eyes closed condition (*t*_(13)_ = 2.498, *p =* .027) over parietotemporal regions (illustrated in Fig 5B). With regard to midline regions, no significant changes were found for the delta or theta frequency bands. Regarding the alpha frequency band, post-hoc tests revealed an increase in power at midline regions (EC: *t*_(13)_ = -2.332, *p* = .036) in the eyes closed condition, as depicted in Fig 5A. Similar results were found for the eyed open condition (EO: *t*_(13)_ = -1.809, *p* = .093), although not significant at the *α* = 0.05 level.

#### 3.1.3 Relationships between changes in resting state EEG and psychopathy

Investigating the changes in resting state EEG measures in relation to the facets of psychopathy showed interesting results: the lower the subjects scored on the *Lifestyle Facet* (EO: *r* = -.770, *p* = .001) and the *Antisocial Facet* (EO: *r* = -.594 *p* = .025), the more they could increase midline alpha band power over the course of SCP training. No significant relationships regarding the *Psychopathy Total* score or the *Affective* or *Interpersonal Facets* were found.

The opposite pattern was found for the slower frequencies: the reduction in delta band activity at parietotemporal sites was significantly correlated with the PCL-R *Total Psychopathy* score (EC: *r* = -.566, *p* = .035) and the *Affective Facet* (EC: *r* = -.671, *p* = .009). These relationships indicate that the higher the subject’s score on psychopathy (especially a greater lack of empathy and remorse, and shallower affect), the more the activity in the delta frequency band was reduced from before to after SCP-training.

The analyses of changes in the theta frequency band support this view: the higher the subjects scored in *Total Psychopathy* (PCL-R Total Score), the greater was the reduction also in theta frequency band activity at parietotemporal sites from before to after neurofeedback training (EO: *r* = -.579, *p* = .03). No significant relationships regarding the other facets of the PCL-R were found in the present data.

#### 3.1.4 Relationships between changes in resting state EEG and SCP

Regarding SCP differentiation, significant relationships were found between the SCP feedback coefficient and changes in alpha band activity at midline sites (EO: *r* = 0.633, *p* = .015), indicating that a more pronounced training performance (an increase in differentiation between negativity and positivity trials) during the feedback block over time was related to an increase in the alpha band.

**Fig 6:**
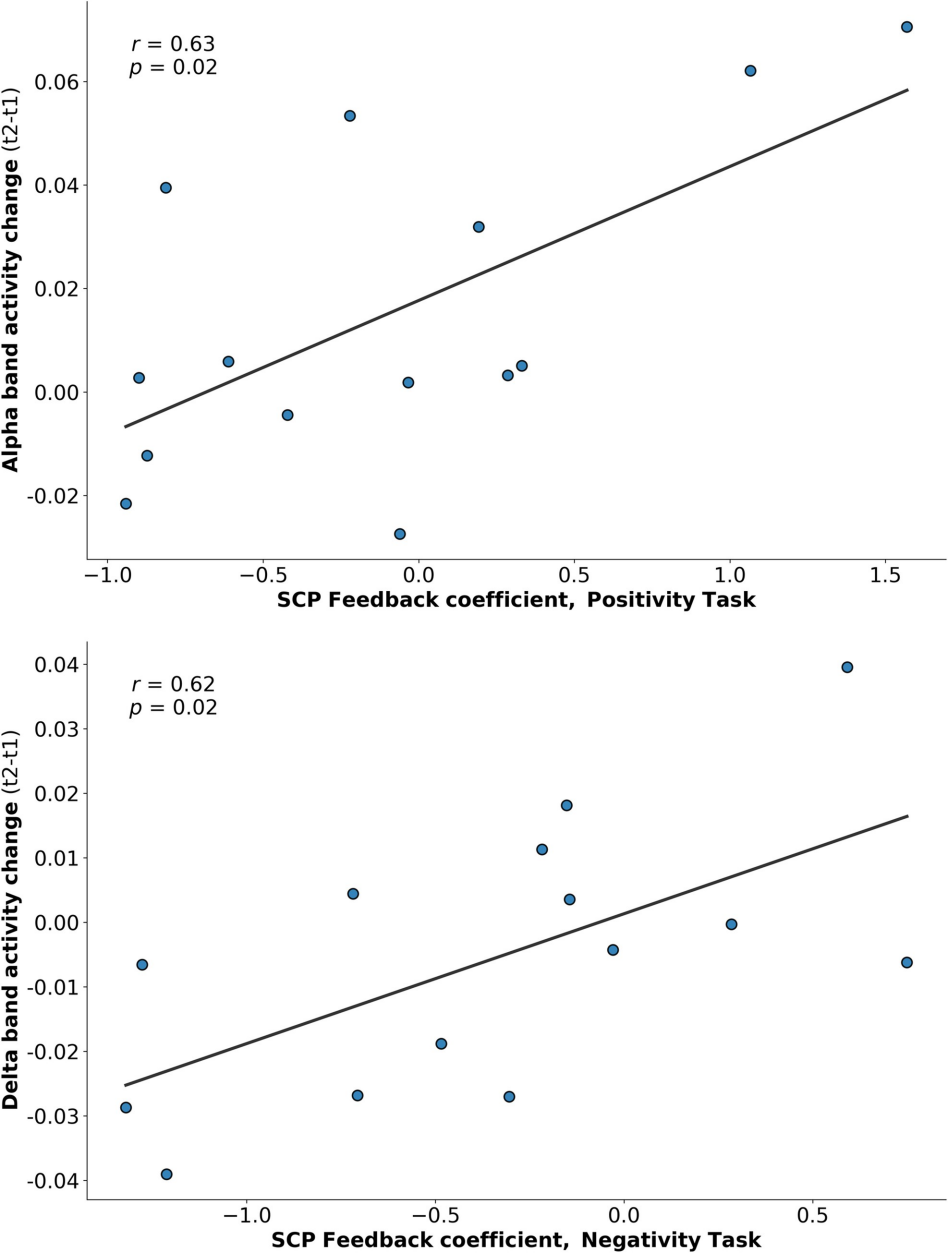
**(a)** Correlation between the increase of alpha band activity and the increase of the volitional production of electrical positive SCP shifts in neurofeedback training. Each dot represents one participant. [X-axis: The higher the positive number, the higher the increase in SCP feedback coefficient in positivity task over time; Y-axis: The higher the positive number, the more increase in alpha band from before to after SCP neurofeedback training]. **(b)** Correlation between the decrease of delta band activity and the increase of the volitional production of electrical negative SCP shifts in neurofeedback training. Each dot represents one participant. [X-axis: The higher the negative number, the higher the increase in SCP feedback coefficient in negativity task over time; Y-axis: The higher the negative number, the more reduction in delta band from before to after SCP neurofeedback training, i.e. negative values toward right].

As depicted in Fig 6A, significant relationships between the increase in alpha band activity at midline regions and especially the SCP coefficient for the positivity trials over time were found for the feedback block (EO: *r* = 0.629, *p* = .016) and the transfer block (EO: *r* = 0.719, *p* = .004). The same picture appeared at parietotemporal regions, showing significant relationships between the increase in alpha band activity and the SCP positivity coefficient in both, the feedback (EO: *r* = 0.593, *p* = .025) and the transfer condition (EO: *r* = 0.704, *p* = .005).

Regarding the delta frequency band, a significant relationship was found between the decrease in delta band activity from before to after neurofeedback training at midline regions and the SCP negativity coefficient (EC: *r* = 0.619, *p* = .018). As illustrated in Fig 6B, this correlation demonstrates that the more participants were able to increase the electrical negative SCP-shift from the beginning to end of the SCP neurofeedback training, the greater was their reduction in delta frequency activity. In order to account for the effects of possible outliers in the training data, a Bonferroni outlier test for linear models and an inspection of the training data via boxplot was performed. Both methods revealed no statistical outliers in the training data. For changes in SCP from the beginning to the end of the training, see Konicar et al. [59].

#### 3.2 Electrodermal activity: Pre-post measures (t2-t1)

**Fig 7:**
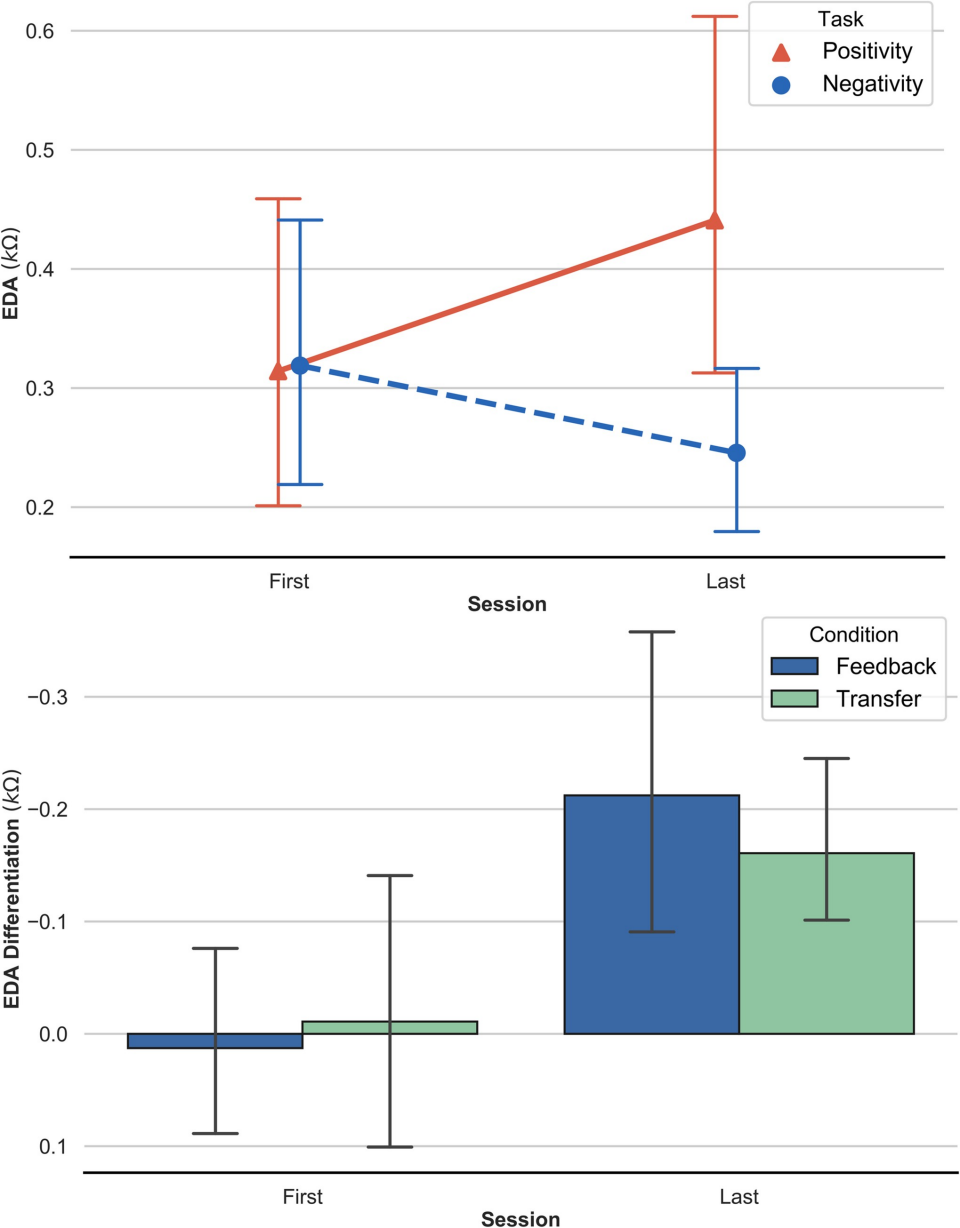
a. Time-Task-Interaction: Average electrodermal response e.g. electrodermal activity (EDA) over the first and the last six SCP-training sessions in the positivity task (red) and the negativity task (blue). **b.** Average electrodermal response e.g. electrodermal activity (EDA): EDA discrimination over the first and the last six SCP-training sessions. The figure shows the strength of the effect in the two different conditions (feedback vs. transfer).

A repeated-measures ANOVA of EDA data revealed a significant main effect of *‘Task’* (*F*_(1,13)_ = 7.85, *p =* .01, *η* = 0.041), and a significant interaction between *‘Time’* and *‘Task’* (*F*_(1, 13)_ = 18.34, *p =* .0009, *η* = 0.045), with reported effect sizes corresponding to the generalized eta squared metric. These results are presented in Fig 7A.

In addition, we performed a Bayesian ANOVA to directly quantify the relative preference of the model including the relevant interaction (*‘Time’* and *‘Task’*) over the model without interaction (i.e., only main effects). Indeed, the resulting Bayes factor in favor of the interaction model was estimated at *BF* = 27.37, which supports the inclusion of the interaction term.

Pairwise *post-hoc* tests adjusted with Tukey’s correction method showed a significant increase in EDA activity from the first six sessions (*M* = .31, *SE* = .06) to the last six sessions (*M* = .44, *SE* = .06) in the positivity task (*t*_(13)_ = -3.565, *p =* .008) (requiring electrically positive SCP shifts, e.g. decreased cortical preparation), as depicted in Fig 7A. The corresponding Bayes factor in favor of the target hypothesis was *BF* = 33.61, suggesting strong evidence for an increase in total EDA. The opposite trend was observable in the negativity task (requiring electrically negative SCP shift, that is, increased cortical preparation), but did not reach statistical significance (the corresponding Bayes factor was *BF* = 1.02, corroborating the frequentist analysis). Further, there was a significant difference in EDA in the last sessions (*t*_(13)_ = 4.734, *p =* .0005), *BF* = 11,79, displaying higher skin resistance (*M* = .44, *SE* = .06) (e.g. less autonomous cholinergic arousal) during positivity tasks than during negativity tasks (*M* = .25, *SE* = .06). This indicates that the subjects EDA discrimination (between positivity and negativity tasks) increased towards the end of SCP training.

Regarding changes in EDA discrimination between positivity tasks and negativity tasks, a significant main effect of *‘Time’* emerged (*F*_(1, 13)_ = 15.36, *p =* .002) confirming an increase in psychophysiological EDA discrimination in both feedback conditions over time (see Fig 7B). The Bayes factor in favor of the model with a single main effect of *‘Time’* against the model including a main effect of *‘Condition’* and an interaction between *‘Time’* and *‘Condition’* was estimated at *BF* = 8.4, supporting an overall increase in discrimination.

Note, that in all EDA analyses, Bayes factors regarding quantities of interest are relatively small, reflecting the large epistemic uncertainty due to the small and heterogeneous sample.

Finally, no relationships emerged between the indices of EDA and the indices of resting state EEG, SCP training coefficients or psychopathy indices. For exploratory analysis regarding relationships between changes in EDA and changes in self-reported empathy, aggressiveness and behavioral approach, see S1-S4 Section in S1 File.

## 4 Discussion

The present study presents additional data and analyses from a previous brain regulation intervention project with incarcerated offenders with psychopathy based on the PCL-R. Complementary to the previously published results (see [59]), the current analyses aimed to investigate within-subject resting-state EEG and electrodermal activity during SCP as well as their relationships to SCP training indices and subjective measures. The analyses presented here are exploratory in nature and thus aim at pointing future research directions instead of confirming specific hypotheses.

Regarding the baseline resting state EEG of these offenders with psychopathy, our results are suggestive of an association between higher baseline power of delta frequencies and higher *Psychopathy Total* scores. Moreover, we found a relationship between the *Interpersonal Facet* of psychopathy and increased delta activity. Considering the whole body of research linking mostly antisocial features (and therefore more the factor 2 of the PCL-R) to slow wave EEG activity, this result is quite surprising, indicating that slow wave activity (like delta and/ or theta activity) is not only a marker for aggressiveness, impulsivity, risk taking and antisocial behavior. Indeed, our results suggest that the pronounced presence of slow wave activity is also linked to core (F1) psychopathy features, like the psychopathy-related superficial charm and manipulation ability, similar to the findings of Calzada-Reyes and colleagues [15].

Still, it is unclear whether this underarousal should be interpreted as a consequence of specific brain abnormalities or due to the effects of chronic underarousal related to incarcerated samples, for instance, in forensic institutions or a general cortical underarousal, that is, irregular brain development / maturity. In contrast to Calzada Reyes and colleagues [15], we found that reduced alpha power at baseline was not related to higher scores on the *Affective Facet* of the PCL-R, but rather to higher scores in the *Lifestyle Facet* of the PCL-R. This could be explained by EEG findings of individuals in the ‘impulsivity spectrum’. The latter includes a wide range of disorders, not only antisocial, psychopathic personality and conduct disorders, but also substance dependencies, pyromania, pathological gambling and other disinhibitory disorders [69]. Since low alpha band activity is a consistently reported biomarker in these externalizing disorders [70, 71], it fits to our finding of an attenuated alpha band at baseline as being related to the *Lifestyle Facet* of the PCL-R, which contains items such as need for stimulation, lack of realistic long-term goals, irresponsibility and impulsivity.

Regarding possible within-subject changes in resting state EEG before compared to after the brain regulation intervention, our analyses suggest that active training of brain regulation in offenders with psychopathy can induce observable changes in the central nervous system. Indeed, our results indicate that resting state EEG patterns do change after EEG neurofeedback training, in terms of a significant reduction of slow frequencies (e.g. a decrease in delta and theta frequency activity), parallel to an increase in alpha band activity. Therefore some further evidence could be added to the tendencies, found in the rare literature on resting state EEG pre/post SCP neurofeedback training, pointing towards theta and delta reductions and/or alpha increases [72, 73].

Regarding the relationship between different psychopathic features (facets of the PCL-R) and electrophysiological parameters, our exploratory analyses hint at two effects. Firstly, alpha frequency increases were diminished for those subjects scoring high on especially antisocial lifestyle characteristics. Secondly, those offenders who scored high on affective deficits and *Psychopathy Total* seem to exhibit even greater reductions of slow frequencies after SCP-neurofeedback training.

To extend the existing literature regarding the link between pre-/post- changes in EEG and different neurofeedback trainings [72–76], we conducted detailed analysis regarding the tasks of SCP-neurofeedback training and EEG resting state changes. Our analysis suggests that the increased production of electrically positive SCP shifts is related to an increase in alpha frequency band. The opposite pattern was found for the delta frequency band: increased electrically negative SCP-shifts were associated with reductions in the delta activity band, which supports a possible ‘balancing’ effect of SCP-neurofeedback on cortical parameters of these individuals with psychopathy.

The relationships between peripheral physiological parameters and SCP-neurofeedback training have not been systematically investigated before. The exploration of possible relationships between central and autonomic functions revealed an increase in discrimination of electrodermal response, in parallel to the increase of reported [59] SCP-differentiation. In other words, participants increased their skin resistance (i.e., decreased autonomic arousal) during the production of electrically positive SCP shifts and also increased their discrimination in EDA between the positivity task and the negativity task over the course of the SCP neurofeedback training.

Albeit only correlational, the current results are suggestive of an underlying biological mechanism relating the behavioral factors of psychopathy to resting state EEG oscillations. At the same time, the results indicate that neurofeedback training of slow cortical potentials may have a frequency-normalizing effect on resting state EEG and thus affects not only behavior or event-related potentials as reported before [59], but possibly the neurophysiological “fingerprint” of the psychopathic trait. Moreover, our results point towards the adaptive nature and flexibility of peripheral body functions in patients with psychopathy and merit further investigation.

This study includes a very rare sample of severe offenders with psychopathy, requiring challenging recruitment, clinical assessment and extensive brain self-regulation training with high security conditions in a forensic institution. Nevertheless, proof-of-concept approaches with small sample sizes like in the current study do not fulfill elaborate neurofeedback design requirements (see Strehl et al. [77]). Potential influences, such as placebo response (including participant expectation), demand characteristics, and spontaneous remission, summarized here as nonspecific treatment factors, as well as specific effects from the neurofeedback treatment procedure could have biased the results [78]. These shortcomings hinder the demonstration of clinical effects and outcomes, not only due to the limited (but at the same time, reassuring) access to highly offenders with psychopathy in Europe and elsewhere (for a further discussion see S5 Section in S1 File).

Therefore, caution regarding further far-reaching speculations and interpretations of our data is recommended. However, our current work follows the idea of an EEGCopia [79] to foster and coordinate results regarding causal and engineerable EEG biomarkers for mental disorders using fine-grained analyses of brain-regulation over time (targeting a better comprehension of the learning processes underlying the EEG neurofeedback procedure) as well as explore relationships between peripheral-physiological and cortical outcomes. Therefore, we urge future research to address important research gaps (by e.g. multicenter data collection) and elucidate, by means of functional neuroimaging, the dynamic neural signature of psychopathy and investigate its underlying relationship with empathy, aggression, and neural plasticity. In a possible next step, future research should evaluate the utility and effects of various neurobiologically plausible treatments and explore novel approaches combining the advantages from EEG and MRI [80, 81], applicable to offenders with psychopathy placed in high security institutions.

## Supporting information

S1 File (DOCX)
