# Supplementary material for: Balancing the brain of offenders with psychopathy? Resting state EEG and electrodermal activity after a pilot study of brain self-regulation training
Source: PLoS One. 2021 Jan 7;16(1):e0242830. doi: 10.1371/journal.pone.0242830 (PMC7790284; doi:10.1371/journal.pone.0242830)
Supplement: S1 File — (DOCX) [file pone.0242830.s001.docx]

**Content**

1. Measures of behavioral approach, aggression and empathy 2

(Self-reports: BPAQ, BISBAS and IRI)

1. Measures of heart rate and heart rate variability 3
2. Statistical analysis of self-report measures and heart rate measures 4
3. Results 5

4.1 Changes in self-reported empathy, behavioral approach and aggression (t_2_-t_1_) 5

4.2 Changes in heart rate and heart rate variability during SCP training (t_2_-t_1_) 7

4.3 Relationships between changes in resting state EEG and changes in self-reports 9

4.4 Relationship between changes in EDA and changes in self-reports 12

4.5 Relationships between psychopathy scores and changes in self-reports 13

1. Design of clinical effect studies in psychopathic offenders 15
2. References 17
3. Measures of empathy, behavioral approach and aggression **(Self-reports: BPAQ, BISBAS and IRI)**

In an exploratory manner, we investigated self-reported empathy from before and after SCP-neurofeedback training. For assessing empathic competencies, the German version of the Interpersonal Reactivity Index (IRI; German version: Saarbrücker Persönlichkeitsfragebogen SPF [1]; Paulus [2]) was used. Besides the Total IRI Score, the IRI encompasses four different dimensions of dispositional empathy: (a) Fantasy, (b) Empathic Concern, (c) Distress and (d) Perspective Taking, showing good psychometric properties (re-test reliability between .62 and .80 [3]).

In addition to the already published results of the main study of Konicar et al [4], showing a decrease in behavioral approach and aggression, relationships between those self-reports and physiological indices were analyzed. Thus, a brief description of the measures is provided as follows.

The Behavior-Inhibition/Behavior-Activation System Questionnaire (BIS/BAS [5]) comprising a BIS Total Score and a BAS Total Score (containing the subscales BAS Drive, BAS Fun Seeking and BAS Reward Responsiveness) was given to the participants to detect behavioral approach or avoidance tendencies.

The German version of the Buss Perry Aggression Questionnaire (BPAQ [6]) was used as an index for aggression. Besides a Total Score it includes the subscales (a) Physical Aggression, (b) Verbal Aggression, (c) Anger and (d) Hostility.

The used self-report questionnaires show satisfying re-test reliability: BPAQ [6] between 62 – .81 and BISBAS [7] between 0.59-0.69.

# Measures of heart rate and heart rate variability

Attenuated resting heart rate is known to be a robust physiological correlate of antisocial behavior [8-20] and was found to be a general risk factor for proactive aggressive and impulsive psychopathic behavior in childhood and adolescence, as well as criminal behavior in adulthood [15-17]. In adults, studies show decreased heart rate in individuals with psychopathic traits compared to controls during cognitively demanding or stressful tasks [12-14].On the other hand, some studies find no associations between heart rate measures and psychopathy or antisocial behavior [18-20].

Therefore we aimed to explore this psychophysiological parameter in our psychopathic sample in an explorative manner, which is described as follows.

Heart rate was recorded using a finger pulse oximeter, a component of the Theraprax Q-EEG-System (NeuroConn GmbH, Illmenau, Germany), during the whole SCP-neurofeedback training. The Pan-Tompkins algorithm [21] was applied to detect QRS complexes in the raw heart rate signal, which was previously high-pass filtered at 0.01 Hz. Based on the approach of Vollmer [22], we quantified heart rate variability via the *rMSSD* indicator:

|  | $RMSSD= \sqrt{\frac{1}{n-1}\sum_{i=1}^{n-1} \left( {RR}_{i}- {RR}_{i+1} \right)^{2}}$ |  |
| --- | --- | --- |

The *rMSSD* gives the root mean square difference between successive R-R intervals and is considered a reliable and valid time-domain measure of heart-rate variability. Heart rate data was segmented into trials and averaged across sessions in a similar manner as with electrodermal response (e.g. electrodermal activity, EDA) data. The time window for calculating the *rMSSD* was 200 (20x10) seconds for the positive and 200 (20x10) seconds for the negative trials during the first 6 sessions and 80 (8x10) seconds for the positive and 320 (32x10) seconds for the negative trials during the last 6 sessions. Data for the first 6 and last 6 sessions were averaged separately.

# Statistical analysis of self-report measures and heart rate measures

In order to investigate changes in the IRI in an exploratory manner, paired sample *t*-tests were performed. Regarding the exploratory inspection of relationships between self-report scores, resting state EEG and EDA, scores from pre-measures were subtracted from post-measure scores (t2-t1) and used as change indices in addition to the reported neurophysiological indices of SCP training^4^.

The statistical analysis of and heart rate variability was done with a 2x2x2 repeated measures ANOVA with factors *“Time”* (first six training sessions / last six training sessions), *“Condition”* (feedback blocks / transfer blocks) and *“Task”* (positivity trials/ negativity trials).

For all exploratory analyses of EEG and peripheral physiological measures, two-tailed p-values were used; no Bonferroni correction was applied due to the exploratory nature of the analyses.

The Greenhouse Geisser Correction was applied whenever the sphericity assumption was violated.

# 4. Results

4.1 Changes in self-reported empathy, behavioral approach and aggression (t2-t1)

In addition to the reported improvements in aggression (BPAQ) and approach behavior (BISBAS) after SCP neurofeedback training^51^, changes in empathy were detected. While the increase in the Total IRI did not reach the level of significance, both the subscale Perspective Taking (*t*_(13)_= -1.932, *p* = .038) and the subscale Empathic Concern (*t*_(13)_= -2.28, *p* = .02) increased significantly from before to after SCP-neurofeedback training. An overview over all self-reported changes is summarized in Table 1.

**Table 1. Changes in self-reports.** (BPAQ: Buss-Perry Aggression Questionnaire, BISBAS: Behavioral Approach Behavioral Avoidance, IRI: Interpersonal Reactivity Index). t_1_= before brain self-regulation training, t_2_ = after brain self-regulation training.

.

| ***IRI*** | ***Mean (t1)*** | ***SD(t1)*** | ***Mean (t2)*** | ***SD (t2)*** | ***T (t2)*** | | ***P*** (t_2_) |
| --- | --- | --- | --- | --- | --- | --- | --- |
| **Fantasy** | 12,29 | 2,894 | 11,57 | 3,589 | ,801 | | 0,219 |
| **Perspective Taking** | 14,00 | 3,783 | 15,14 | 2,770 | -1,932 | | **0,038*** |
| **Empathic Concern** | 14,14 | 2,538 | 15,57 | 1,651 | -2,280 | | **0,020*** |
| **Distress** | 9,00 | 2,353 | 8,71 | 2,268 | ,409 | | 0,345 |
| **IRI Total** | 31,43 | 7,035 | 33,57 | 6,607 | -1,226 | | 0,121 |
| ***BISBAS*** | ***Mean*** (t_1_) | ***SD***(t_1_) | ***Mean*** (t_2_) | ***SD*** (t_2_) | ***T*** (t_2_) | | ***P*** (t_2_) |
| **BAS Drive** | 2,98 | 0,55 | 2,79 | 0,64 | 2,24 | **0,022*** | |
| **BAS Fun Seeking** | 3,02 | 0,56 | 2,93 | 0,60 | 1,00 | 0,168 | |
| **BAS Reward Responsiveness** | 3,11 | 0,44 | 2,83 | 0,48 | 2,74 | **0,008*** | |
| **BIS Total** | 2,77 | 0,25 | 2,65 | 0,22 | 1,30 | 0,108 | |
| **BAS Total** | 3,04 | 0,39 | 2,85 | 0,42 | 3,31 | **0,003*** | |
| ***BPAQ*** | ***Mean*** (t_1_) | ***SD***(t_1_) | ***Mean*** (t_2_) | ***SD*** (t_2_) | ***T*** (t_2_) | ***P*** (t_2_) | |
| **Physical Aggression** | 27,00 | 10,08 | 23,86 | 8,42 | 1,995 | **,034*** | |
| **Verbal Aggression** | 14,57 | 3,08 | 14,93 | 4,12 | -,510 | ,310 | |
| **Anger** | 18,86 | 6,05 | 17,79 | 6,68 | ,845 | ,207 | |
| **Hostility** | 21,00 | 5,12 | 21,71 | 4,46 | -,629 | ,270 | |
| **BPAQ Total** | 81,43 | 21,00 | 78,29 | 19,56 | ,903 | ,192 | |

## **4.2 Changes in heart rate and heart rate variability during SCP** **neurofeedback (t2-t1)**

Regarding heart rate and heart rate variability, there were neither significant main effects of ‘*Block*’, nor main effects of *‘Time’* e.g. no significant changes in heart rate or heart rate variability, from the beginning to the end of the SCP-neurofeedback training or between the feedback or transfer condition. A significant main effect of ‘*Task’* was observed (*F*_(1, 13)_ = 17.31, *p* < .001), indicating that heart rate during negativity trials (e.g. increased cortical preparation) was significantly higher than during positivity trials (e.g. decreased cortical preparation). Further, this discrimination was apparent already in the very beginning of brain regulation training and remained stable until the end of the training, as depicted in Fig 1.

In summary, this finding indicates that similarly to the EDA, the heart rate of psychopathic subjects varied according to the different required brain activity levels: heart rate rose during the increase in cortical activation (negativity task), and decreased in trials which required a reduction in cortical activation (positivity task).

Heart Rate Variability (HRV) did not change significantly from the beginning to the end of the SCP neurofeedback training, as indicated by Fig 2.


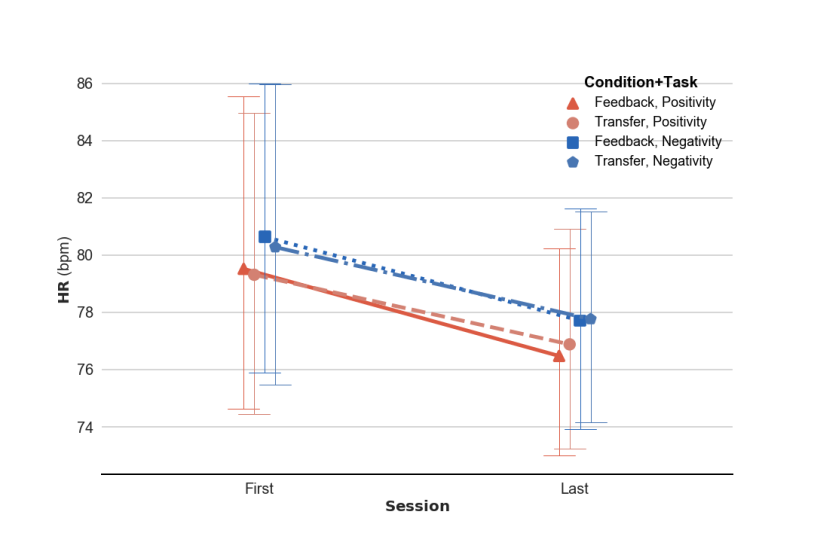


**Fig 1.** **Main effect of SCP neurofeedback task** indicates lower heart rate during positivity tasks of SCP neurofeedback than during negativity tasks of SCP neurofeedback. No main effect of *Time* from the first six to the last six SCP neurofeedback sessions was found.


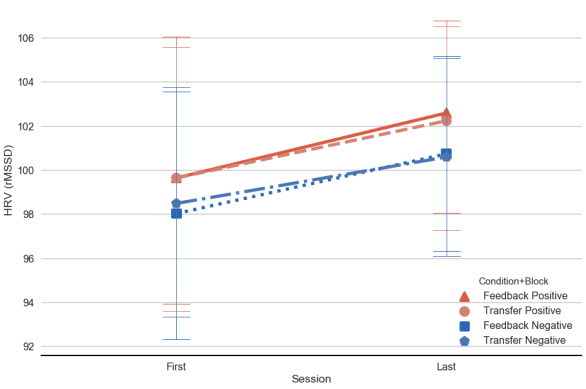


**Fig 2. HRV during SCP-neurofeedback**. Non-significant increase of heart rate variability from the beginning to the end of the SCP neurofeedback training. The significant effect of *Task* indicates higher heart rate variability during positivity trials of SCP-neurofeedback than during negativity trials of SCP-neurofeedback.

## 4.3 Relationships between changes in resting state EEG and changes in self-reports

A significant relationship was found between the subscale Anger of the BPAQ [5] and the change in alpha frequency band at midline regions, indicating, that the more alpha frequency has increased after neurofeedback training, the more the subscale Anger decreased (EC: *r* = -0.62, *p* = .0017) as depicted in Fig 3a.

Theta frequency band changes at parietotemporal regions were found to be moderately correlated with the IRI Total [2] (EC: *r* = -0.54, *p* = .049, see Fig 3b), the subscale Fantasy (EC: *r* = -0.58, *p* = .028, EO: *r* = -0.54, *p* = .047) and the subscale Distress (EC: *r* = 0.52, *p* = .056; EO: *r* = 0.57, *p* = .035). Analysis regarding theta frequency band changes at midline regions are consistent with this pattern: a moderate correlation were found with the IRI subscale Fantasy (EO: *r* = -.57, *p* = .034). These correlations suggest that the more theta frequency band activity decreased at parietotemporal sites, the more Empathy increased from pre to post neurofeedback training. The opposite pattern is observed for Distress: the more theta frequency band activity was reduced after neurofeedback training, the more Distress also decreased.


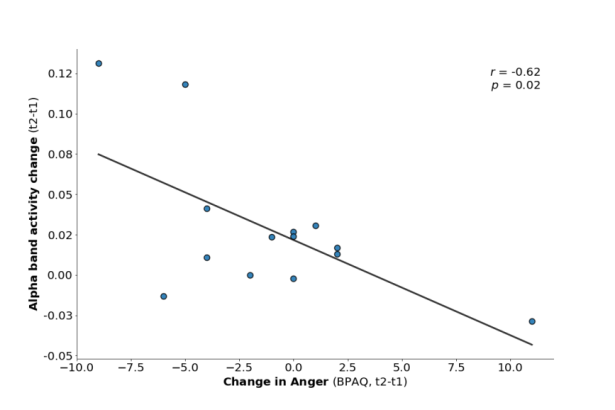


**Fig 3a. Relationship** between the increase of alpha band activity from t1 to t2 and the decrease of Anger (BPAQ; t2-t1). Each dot represents one participant.


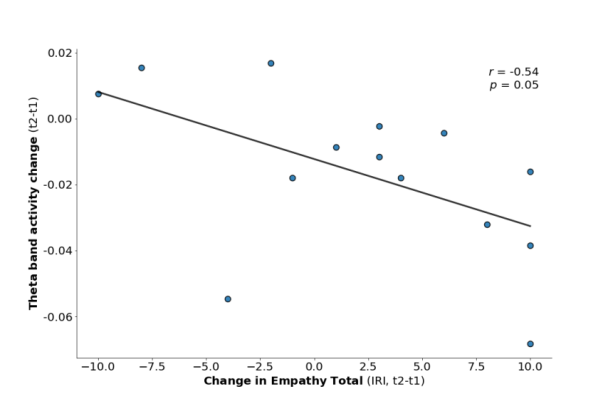


**Fig 3b. Relationship** between the decrease of theta band activity from t1 to t2 and the increase in Empathy Total (IRI) from t1 to t2. Each dot represents one participant.

No significant relationships were found between other psychological self-reports and EEG resting state indices; trends are summarized in the following paragraph.

A trend for a relationship between the increase in alpha frequency band from before to after neurofeedback training at midline regions and the decrease in the subscale Distress of the IRI was found (EC: *r* = -0.49, *p* = .074). Further trends were found between the increase in alpha frequency band from before to after neurofeedback training at parietotemporal regions and the subscale empathic concern (EC: *r* = 0.48, *p* = .079) as well as with the total empathy index IRI Total was found (EC: *r* = 0.48, *p*= .080).

Regarding delta frequency band, no relationship reached the level of significance - only trends with psychological indices were found. Firstly, a trend between the decrease in delta frequency band from before to after neurofeedback at parietotemporal region and the BAS Total Score (EC: *r* = -0.51, *p* = .065) as well as with the BAS subscale Reward Responsiveness (*r* = -0.49, *p* = .074) was found. These relationships suggest that the more delta band is decreasing from before to after neurofeedback training, the less of a decrease is observed in BAS Total. Secondly, trends were found suggesting a relationship with the empathy indices of the SPF. Trends were found between the decreases in delta frequency band at parietotemporal region with the subscale Distress (EC: *r* = 0.49, *p* = .078), as well as with the SPF Total Score (EC: *r* = -0.46, *p* = .095). These trends indicate that the more delta frequency band is decreasing from before to after neurofeedback training, the more the total empathy index is increasing and the more the subscale Distress is decreasing.

In summary, the most interesting finding of these exploratory investigations is that the post-neurofeedback decrease in slow frequency bands was linked to a significant increase in empathy, while the increase in alpha frequency band was found to be related to the reduction in anger. Considering that the two core social emotions: empathy (inversely linked to goal-directed aggression) and anger (linked to reactive aggression) both modulate the risk for aggression [23], the results of the exploratory analyses indicate that both types of aggression, which are traits of psychopathy, are not necessarily permanent, but are potentially modifiable via the a reduction of anger and an enhancement in empathic competencies.

## 4.4 Relationship between changes in EDA and changes in self-reports

The electrodermal response e.g. electrodermal activity (EDA) in negativity trials was found to be linked to the behavior activation system (BAS; The Behavior-Inhibition/Behavior-Activation System Questionnaire, BIS/BAS [5]), i.e. the BAS subscale Reward Responsiveness (*r* = .53, *p* = .05) - supported by a positive trend for a relationship with BAS Total Score (*r* = .48, *p* = .082). These relationships indicate that a decrease in skin resistance (e.g. an increase in peripheral arousal) in negativity trials is accompanied by a decrease in reward and approach behavior over time.

No significant associations were found between the reported [4] changes in the subscale Physical Aggression of the BPAQ [6] following SCP neurofeedback training and changes in EDA. Only a trend for a relationship between EDA and BPAQ Total Score was observed (*r* = .48, *p* = .083), indicating that a decrease in Total Aggression is associated with a decrease in skin resistance (e.g. peripheral arousal is increasing) in negativity trials over time. No significant relationships were found between EDA- indices and changes in IRI [2].

In summary, these exploratory analyses give us a hint that there might be a possible link between self-reported changes in the behavioral activation system (e.g. reductions in approach and reward behavior) and the increased peripheral arousal in the negativity tasks of the SCP-training.

## 4.5 Relationships between psychopathy scores and changes in self-reports

**Table 2. Relationship between psychopathy scores and changes in self-reports.**

Changes of the *empathy indices of the IRI:* the significant relation between the PCL-R Total score and the changes on the subscale Fantasy and similar trends of other SPF – PLC-R subscale, indicate that subjects with higher degrees in psychopathy tend to show also higher increases in empathy indices of the SPF from before to after neurofeedback training than subjects with lower degrees in psychopathy.

Changes in the *behavioral systems of the BIS/BAS:* these correlations indicate that the lower the subject scored on the PCL-R, the higher was the reduction in behavioral approach and avoidance from before to after neurofeedback training.

Changes in *aggression indices of the BPAQ:* the correlations follow a similar trend: the lower the subjects score on the PCL-R, the more the subjects could reduce the different indices of aggression. The only significant exception here is the subscale Hostility: the higher the subjects scored on the PCL-R Lifestyle Facet or on the Facet Antisociality, the more they could reduce Hostility from before to after neurofeedback training.

In the first lines, the correlation coefficient *R* is presented. The second line depicts the *P*-value of the correlation.

| **Changes in self-reports** _(t2-t1)_ | **PCL-R**  **Total Score** | **Interpersonal Facet**  **(PCL-R)** | **Affective Facet (PCL-R)** | **Lifestyle Facet (PCL-R)** | **Antisocial Facet (PCL-R)** |
| --- | --- | --- | --- | --- | --- |
| **IRI subscale Fantasy** | **.587***  **0.027** | -0,084  0,776 | **0,492**  **0,074** | 0,187  0,523 | 0,372  0,191 |
| **IRI Perspective Taking** | 0,126  0,667 | 0,042  0,886 | 0,048  0,872 | 0,409  0,146 | -0,058  0,844 |
| **IRI Empathic Concern** | -0,004  0,990 | -0,199  0,496 | 0,301  0,295 | 0,386  0,172 | -0,196  0,501 |
| **IRI Distress** | -0,147  0,617 | 0,178  0,543 | -0,263  0,363 | -0,368  0,195 | 0,041  0,890 |
| **IRI Empathy** | 0,402  0,154 | -0,118  0,689 | 0,443  0,113 | 0,439  0,116 | 0,118  0,689 |
| **IRI Total Score** | 0,400  0,157 | -0,171  0,559 | **0,481**  **0,082** | **0,520**  **0,057** | 0,083  0,777 |
| **BAS Drive** | 0,297  0,302 | 0,231  0,427 | 0,269  0,352 | 0,259  0,371 | -0,204  0,484 |
| **BAS Fun Seeking** | 0,264  0,363 | -0,017  0,953 | **,566***  **0,035** | 0,339  0,236 | -0,320  0,264 |
| **BAS Reward Responsiveness** | **,827****  **0,000** | 0,310  0,280 | 0,439  0,117 | **,581***  **0,029** | 0,198  0,498 |
| **BIS TOTAL Score** | ,**819****  **0,000** | 0,308  0,285 | **,684****  **0,007** | ,670**  0,009 | -0,108  0,713 |
| **BAS TOTAL Score** | **,787****  **0,001** | 0,295  0,305 | **,694****  **0,006** | **,657***  **0,011** | -0,150  0,609 |
| **BPAQ Physical Aggression** | 0,324  0,259 | 0,395  0,162 | 0,365  0,200 | -0,163  0,577 | -0,309  0,283 |
| **BPAQ Verbal Aggression** | **0,489**  **0,076** | 0,329  0,251 | 0,341  0,233 | -0,065  0,826 | 0,135  0,646 |
| **BPAQ Hostility** | -0,097  0,741 | -0,025  0,934 | 0,012  0,968 | **-,549***  **0,042** | -0,018  0,951 |
| **BPAQ Total Score** | **,541***  **0,046** | 0,131  0,654 | **,818****  **0,000** | -0,040  0,892 | -0,292  0,311 |

# 5. Design of clinical effect studies in psychopathic offenders

From a methodological point of view, in general a double (or triple) blind controlled design is requested to prove clinical effectivity. In such a design matched participants would receive (a) a psychological/behavioral treatment or (b) psychophysiological feedback based on another EEG parameter, like alpha activity; or sham SCP-feedback or feedback from electromyographical activity, as a control condition. For sure a highly comparable control group (i.e., sham neurofeedback) could have revealed whether benefits derive from receiving a particular brain signal. The aim of the main study of Konicar et al. [4] was to investigate the self-regulation abilities and related behavioral outcomes in highly psychopathic patients, and not the comparison of neurofeedback with other similar treatments. Thus, because the present study is based on the data of Konicar et al. [4], and focuses on the exploration of possible changes in cortical, peripheral and psychological indices *after* SCP-neurofeedback, the limitations for the present study are the same as for the main results of the SCP study [4] (and therefore similar to the explanation of the limitation in the main Supplement of Konicar et al [4]).

Comparing the training outcome measures with those of a non-treatment group or a control group (a,b), would have required at least an age-, psychopathy- and offence- matched control group, which is not available in the German forensic hospitals. Note that, our sample consisted of offenders with high psychopathy scores and extreme crimes, exceeding markedly the proposed cut-off score [24] for German and European psychopathic samples. The comparison between our highly psychopathic group with severe and multiple offences and a group consisting of participants with less severe offences or low scores on the PCL-R might have revealed specific differences, but cannot be regarded as a matched control group. In addition, blinding of therapists in self-regulation treatment is impossible, because patients and therapists are continuously informed of the achieved brain changes, which leads to conscious or subliminal perception of treatment progress and therefore uncontrolled placebo responses. Although it is not easy to distinguish neurofeedback training effects from mere placebo effects (e.g., see Thibault et al. [25]), previous studies with healthy and psychopathological samples and different types of control procedures have attempted to disentangle such effects [26,27]. The physiological nature of the task, the extensive training time and the neurobiological measure limits the possibilities of placebo response, but certainly cannot exclude them completely.

Whether the modification of the cognitive and emotional behavior measured in this sample is sufficient for the compensation of the emotional and social deficits in psychopathy is an empirical question and needs larger sample sizes, which was outside the scope of the present study.

Besides questionable validity of staff observations inside prisons or high security forensic units (or behavioral tests inside those institutions), the evaluation of behavioral changes and the internalization of proper social rules of conduct after the treatment by psychopathic patients are difficult tasks. Releasing successfully trained/ treated criminal patients and tracking their aggressive behavior or recidivism is out of the question because of ethical reasons and public safety. Only long-term follow ups after legal release from prison or forensic unit may provide an answer to the generalization problem. Most of the participants of our sample committed offences, which are usually punished with lifelong imprisonment or ordered to undergo indefinite time treatment in high security forensic psychiatry units.

1. **References**
2. Davis M H. A multidimensional approach to individual differences in empathy. JSAS Catalog of Selected Documents in Psychology. 1980; 10: 85.
3. Paulus C. Saarbrücker Persönlichkeits-Fragebogen zu Empathie (SPF). 2009. Retrieved February 25, 2011, from: http://bildungswissenschaften.uni-saarland.de/personal/paulus/ empathy/SPF.html.
4. Davis MH & Franzoi SL. Stability and change in adolescent self-consciousness and empathy. Journal of Research in Personality.1991; 25: 70-87.
5. Konicar, et al. Brain self-regulation in criminal psychopaths. Scientific Reports; 2015; 5: 9426. http://dx.doi.org/10.1038/srep09426.
6. Strobel A, Beauducel A, Debender S & Brocke B. Eine deutschsprachige Version des BIS/BAS-Fragebogens von Carver und White. J. Individ. Differ. 2001; 22: 216–27.
7. Herzberg P Y. Faktorstruktur, Gütekriterien und Konstruktvalidität der deutschen Übersetzung des Aggressionsfragebogens von Buss und Perry. J. Individ. Differ. 2003; 24: 311-23.
8. Carver CS & White TL. Behavioral inhibition, behavioral activation, and affective responses to impending reward and punishment: The BIS/BAS Scales. Journal of Personality and Social Psychology. 1994; 67(2): 319-333.
9. Ortiz J & Raine A. Heart rate level and antisocial behavior in children and adolescents: A meta-analysis. J Am Acad Child Adolesc Psychiatry.2004; 43(2): 154-162.
10. Lorber MF. Psychophysiology of Aggression, Psychopathy, and Conduct Problems: A Meta-Analysis. Psychol Bull. 2004; 130: 531–52.
11. Raine A. Biosocial studies of antisocial and violent behavior in children and adults: A review. J Abnorm Child Psychol.2002; 30(4): 311-326.
12. Raine A. Autonomic nervous system factors underlying disinhibited, antisocial, and violent behavior. Biosocial Perspectives and Treatment Implications. Ann. NY. Acad Sci. 1996; 794: 46–59.
13. Hansen AL, Johnsen BH, Thornton D, Waage L, & Thayer JF. Factors of psychopathy, heart rate variability and cognitive function. Journal of Personality Disorders.2007; 21(5): 568-582.
14. Vries‐Bouw, D. et al. The predictive value of low heart rate and heart rate variability during stress for reoffending in delinquent male adolescents. Psychophysiology. 2011; 48(11): 1597-1604.
15. Fairchild G. et al. Cortisol diurnal rhythm and stress reactivity in male adolescents with early-onset or adolescence-onset conduct disorder. Biological Psychiatry.2008; 64(7): 599-606.
16. Raine A, Fung ALC, Portnoy J, Choy O & Spring VL. Low heart rate as a risk factor for child and adolescent proactive aggressive and impulsive psychopathic behavior. Aggressive Behavior.2014; 40: 290-299.
17. Murray J. et al. Low resting heart rate is associated with violence in late adolescence: a prospective birth cohort study in Brazil. Int. J. Epidemiol. 2016; 45(2): 491–500.
18. Raine A, Venables PH & Williams M. High autonomic arousal and electrodermal orienting at age 15 years as protective factors against criminal behavior at age 29 years. American Journal of Psychiatry.1995; 152: 1595–1600.
19. Portnoy J & Farrington D P. Resting heart rate and antisocial behavior: An updated systematic review and meta-analysis. Aggression and violent behavior.2015; 22: 33-45.
20. Kavish, N. et al. Physiological Arousal and Juvenile Psychopathy: Is Low Resting Heart Rate Associated With Affective Dimensions? Psychiatric Quarterly.2017; 88(1): 103-114.
21. Kavish NA, Fu Q, Vaughn M, Qian Z, & Boutwell B. Resting heart rate and psychopathy: Findings from the Add Health Survey. 2018; bioRxiv: 205005.
22. Pan J & Tompkins WJ. A real-time QRS detection algorithm. IEEE Trans. Biomed. Eng. 1985; 32: 230–236.
23. Vollmer M. A robust, simple and reliable measure of heart rate variability using relative RR intervals. In Computing in Cardiology Conference (CinC) IEEE. 2015; 609-612.
24. Blair RJR. Traits of empathy and anger: implications for psychopathy and other disorders associated with aggression. Philos. Trans. R. Soc. Lond. B. Biol. Sci. 2018; 19: 373(1744).pii: 20170155. doi: 10.1098/rstb.2017.0155.
25. Cooke DJ, Hart SD & Michie, C. Cross-national differences in the assessment of psychopathy: do they reflect variations in raters’ perceptions of symptoms? Psychol. Assess. 2004; 16: 335–339.
26. Thibault RT, Lifshitz M, Birbaumer N & Raz A. Neurofeedback, Self-Regulation and Brain Imaging: Clinical Science and Fad in the Service of Mental Disorders. Psychother Psychosom., 2015; 84(8): 193-207.
27. Gevensleben H et al. Distinct EEG effects related to neurofeedback training in children with ADHD: a randomized controlled trial. Int J Psychophysiol. 2009; 74(2): 149-57.
28. Strehl U, Aggensteiner P, Wachtlin D, Brandeis D, Albrecht B, Arana, M. et al. Neurofeedback of Slow Cortical Potentials in Children with Attention-Deficit/Hyperactivity Disorder: A Multicenter Randomized Trial Controlling for Unspecific Effects. Front Hum Neurosci., 2017; 31: 11,135.
